# ﻿Systematics, natural history, and conservation of *Erica* (Ericaceae)

**DOI:** 10.3897/phytokeys.257.159429

**Published:** 2025-06-06

**Authors:** Michael D. Pirie, Anina Coetzee, Jaime Fagúndez, Félix Forest, Seth Musker, E. Charles Nelson, Timotheüs van der Niet, Fernando Ojeda

**Affiliations:** 1 University Museum, University of Bergen, Postboks 7800, N-5020 Bergen, Norway; 2 FitzPatrick Institute of African Ornithology, DSI-NRF Centre of Excellence, University of Cape Town, Rondebosch, 7701, South Africa; 3 Conservation Management Department, Nelson Mandela University, George, 6529, South Africa; 4 Universidade da Coruña, BIOCOST research group, Centro Interuniversitario das Paisaxes Atlánticas Culturais (CISPAC), 15707, Santiago de Compostela, Spain; 5 Universidade da Coruña, Facultade de Ciencias, Departamento de Bioloxía, 15071, A Coruña, Spain; 6 Royal Botanic Gardens, Kew, Richmond, TW9 3AE, UK; 7 Department of Biological Sciences, University of Cape Town, Private Bag, Rondebosch 7701, South Africa; 8 Tippitiwitchet Cottage, 255A Broadgate, Sutton St Edmund PE12 0LT, UK; 9 Centre for Functional Biodiversity, School of Life Sciences, University of KwaZulu-Natal, Scottsville, Pietermaritzburg 3209, South Africa; 10 Departamento de Biología-IVAGRO, Universidad de Cádiz, Campus Río San Pedro, 11510 Puerto Real, Spain; † Deceased

**Keywords:** Biodiversity crisis, Global Conservation Consortium for *Erica*, integrated plant conservation, pollination, species identification, threat status, World Flora Online

## Abstract

Worldwide, many tens of thousands of plant species are threatened with extinction in the wild. Those that are naturally rare or have narrow distributions are particularly vulnerable. The flowering plant genus *Erica* is a prime example, its numerous species presenting additional challenges associated with conserving large, complex, plant groups. This topical collection of papers represents a substantial body of research on inter-dependent themes in nomenclature, taxonomy, phylogeny, ecology, and conservation of *Erica* species. It will serve as a lasting resource and reference, helping us to better understand, describe, and name species diversity and improving our knowledge of the complexity of ecological interactions in the wild. These advances will aid and champion conservation of natural habitats and support targeted efforts to secure the most critically endangered species in seed banks and botanic gardens.

## ﻿

In this topical collection, we present 12 interlinked papers on the nomenclature, taxonomy, phylogeny, ecology, and conservation of species of the large flowering plant genus *Erica* (Ericaceae). The numerous species of *Erica* are concentrated in South Africa’s spectacular Cape Floristic Region (Oliver et al. 1983), but with diversity extending through Africa (Beentje 2006), Madagascar (Perrier de la Bathie 1927), and into Europe (Nelson 2011). Many are naturally rare or have narrow distributions, placing them amongst the sobering proportion of plant species worldwide that are threatened with extinction in the wild (Lughadha et al. 2020). Despite its relatively young age, the genus harbours a tremendous morphological diversity, most notably in floral traits (Rebelo et al. 1985). Apart from pollinator-driven differentiation (e.g. Johnson 1996; Musker et al. 2024), its enormous species richness is associated with the seeder post-fire response in areas with high winter rainfall reliability (e.g. Ojeda 1998; Cowling et al. 2018). The collection includes reviews and syntheses consolidating existing knowledge and making it findable and accessible, as well as original research and novel data. Their collective aims are to better understand, describe, and name *Erica* species diversity, to enable accurate identification, to understand their ecological interactions in the wild, and to support their conservation.

Progress in understanding the evolution of any lineage depends on sound taxonomy, especially for a large genus such as *Erica* that includes groups of species characterised by relatively minor morphological differences, and which has a long history of horticultural innovation with numerous cultivars. The work of Elliott et al. (2024) is fundamental, providing an authoritative reference for *Erica* names through the World Flora Online (WFO), which is regularly updated and maintained by the Taxonomic Expert Network for Ericaceae. During the process of integrating data from the International Register of Heather Names (Nelson and Small 2000, 2004) into WFO, Nelson et al. (2023) clarified longstanding issues in *Erica* nomenclature, replacing 13 illegitimate names for Cape species. In June 2024, WFO listed 851 accepted *Erica* species. Of those, 65% were represented in a phylogenetic analysis by Pirie et al. (2024) used to identify Evolutionarily Distinct and Globally Endangered (EDGE) species (Isaac et al. 2007). The WFO data now forms the taxonomic backbone for a revitalised version 4 of the *Erica* ID aid (Oliver et al. 2024). This, in turn, has been updated to incorporate data on the poorly understood Malagasy *Erica* diversity (Hackel et al. 2025). It has also been used as a morphological comparative tool, in combination with the phylogenetic information provided by Pirie et al. (2024), to assess and describe novel taxa in Cape *Erica* (Hoekstra et al. 2025).

The development of molecular systematics and implementation of novel methods can reveal great insight into species-level systematics. Musker et al. (2025) presented new Ericaceae-specific hybrid sequence capture markers and resources for phylogenomic inference in rapidly diverged lineages and species complexes. Rodríguez-Buján et al. (2024) used a different high-throughput sequencing approach, analysing single nucleotide polymorphisms from shorter RADseq sequences, combined with morphology, to test species boundaries in two European species. They could confirm the status of the narrow endemic *Ericaandevalensis*. Pasta et al. (2025) provided detailed documentation for a further European species: *Ericasicula*, which occurs across the Mediterranean basin, but shows a highly fragmented distribution pattern.

The evolution of floral diversity requires thorough understanding of plant-pollinator interactions. Two works, by McCarren et al. (2024) and by Van der Niet and Cozien (2024), improved our understanding of the complex interactions of *Erica* species with their pollinators. Beyond the field of pollination ecology, this work serves to illustrate the importance of species conservation to preserve ecological interactions in native habitats.

Improving the existing tools for species identification aids in research and public engagement in biodiversity conservation. The *Erica* ID aid will continue to be an essential resource for both amateurs and professionals attempting to identify *Erica* species, and there is a clear route to further improvement. For example, more of the subspecific taxa can be coded, and better distribution and phenology data, as well as documentation of other diagnostic traits, would further enhance its performance (Oliver et al. 2024; Hoekstra et al. 2025). There remains undescribed diversity, even in the already recognised enormous richness of Cape *Erica*, and a substantial shortfall in phylogenetic data for African and Madagascan diversity (both representation of taxa and resolving power of the most widely used markers), without which it is more difficult to identify EDGE species with confidence. Direct evidence for pollinators of *Erica* species is limited. For threat status, we still rely on the foundational work of Raimondo et al. (2009). This now needs updating and expanding, particularly for endangered and data-deficient taxa. The situation described by Hackel et al. (2025) for Malagasy *Erica* represents the starkest knowledge gap: we must resolve uncertainty in species limits and specimen identifications as an urgent prerequisite to formal threat assessments.

Together, this set of papers presents both individual advances and a clearer view of knowledge gaps. We are now in a stronger position to translate existing knowledge and resources into priorities for action, to more effectively obtain and target funding for conservation efforts in natural habitats, and to secure the most critically endangered species in seed banks and botanic gardens (Pirie et al. 2022). We believe that the synergy created through taxon-specific topical collections such as this is hugely beneficial for advancing both systematics and its dependent disciplines and endeavours.

Expanding knowledge of large genera requires dedicated scientists who push the existing boundaries during lifelong carriers. In working towards this collection, our aim was to provide lasting resources and reference for future work, but also to honour the enormous contribution to *Erica* taxonomy of our longstanding collaborator E.G.H. (Ted) Oliver and his late wife Inge M. Oliver. This is described in a dedicated review article by Nelson et al. (2024). Ted sadly passed away in January 2025. He was recently honoured with another species (*Eulophiaedwardii* Bytebier; Bytebier and Grieve 2025) described in Orchidaceae, his other group of special interest. We could also not have anticipated the loss of E. Charles Nelson, both author and editor on this collection, in May 2024. His documentation and clarification of *Erica* nomenclature over many years, as well as his authoritative monograph of the northern species (Nelson 2011), was just part of a remarkable body of work (Shaughnessy 2024) and his productivity showed no signs of slowing. This collection is therefore dedicated with immense gratitude to Ted, Inge, and Charles.

## ﻿Additional information

### Author ORCIDs

Michael D. Pirie https://orcid.org/0000-0003-0403-4470

Anina Coetzee https://orcid.org/0000-0002-1646-557X

Jaime Fagúndez https://orcid.org/0000-0001-6605-7278

Félix Forest https://orcid.org/0000-0002-2004-433X

Seth Musker https://orcid.org/0000-0002-1456-1373

E. Charles Nelson https://orcid.org/0000-0002-3909-4388

Timotheüs van der Niet https://orcid.org/0000-0002-5250-8995

Fernando Ojeda https://orcid.org/0000-0001-5480-0925

### Data availability

All of the data that support the findings of this study are available in the main text.

## References

[B1] Beentje HJ (2006) Ericaceae. In: Beentje HJ Ghazanfar SA (Eds) Flora of Tropical East Africa.Royal Botanic Gardens, Kew, 1–29.

[B2] Bytebier B Grieve KW (2025) 1135. *Eulophiaedwardii* Bytebier: Orchidaceae. Curtis’s Botanical Magazine, 1–8. 10.1111/curt.12624

[B3] Cowling RM Gallien L Richardson DM Ojeda F (2018) What predicts the richness of seeder and resprouter species in fire-prone Cape fynbos: Rainfall reliability or vegetation density? Austral Ecology 43: 614–622. 10.1111/aec.12606

[B4] Elliott AC Bester SP Klopper RR Nelson EC Pirie MD (2024) Curating an online checklist for *Erica* L. (Ericaceae): Contributing to and supporting global conservation through the World Flora Online. PhytoKeys 243: 121–135. 10.3897/phytokeys.243.121555 38947554 PMC11214009

[B5] Hackel J Razafimanantsoa AHI Porcher V Pirie MD (2025) Heathers (Erica, Ericaceae) of Madagascar: Taxonomy, evolution, ecology and uses. PhytoKeys 256: 91–118. 10.3897/phytokeys.256.141375 40438531 PMC12117345

[B6] Hoekstra RD Musker SD Pirie MD Vlok JHJ (2025) An integrative approach to alpha taxonomy in *Erica* L. (Ericaceae) with three new species from the Western Cape, South Africa. PhytoKeys. [in press] 10.3897/phytokeys.257.139457 PMC12159662 40510408

[B7] Isaac NJB Turvey ST Collen B Waterman C Baillie JEM (2007) Mammals on the EDGE: Conservation priorities based on threat and phylogeny. PLoS ONE 2(3): e296. 10.1371/journal.pone.0000296 PMC1808424 17375184

[B8] Johnson SD (1996) Pollination, adaptation and speciation models in the Cape flora of South Africa. Taxon 45: 59–66. 10.2307/1222585

[B9] Lughadha EN Bachman SP Leão TCC Forest F Halley JM Moat J Acedo C Bacon KL Brewer RFA Gâteblé G Gonçalves SC Govaerts R Hollingsworth PM Krisai-Greilhuber I de Lirio EJ Moore PGP Negrão R Onana JM Rajaovelona LR Razanajatovo H Reich PB Richards SL Rivers MC Cooper A Iganci J Lewis GP Smidt EC Antonelli A Mueller GM Walker BE (2020) Extinction risk and threats to plants and fungi. Plants, People, Planet 2: 389–408. 10.1002/ppp3.10146

[B10] McCarren S Midgley JJ Coetzee A Johnson SD (2024) Pollen transfer efficiency in *Erica* depends on type of pollinator. PhytoKeys 244: 237–248. 10.3897/phytokeys.244.107288 39086738 PMC11289515

[B11] Musker S Pirie M Nürk N (2024) Pollinator shifts despite hybridisation in the Cape’s hyperdiverse heathers (*Erica*, Ericaceae). Molecular Ecology 33: e17505. 10.1111/mec.17505 39188071

[B12] Musker S Pirie M Nürk N (2025) Worth the fuss? Maximising informativeness for target capture-based phylogenomics in *Erica* (Ericaceae). PhytoKeys 251: 87–118. 10.3897/phytokeys.251.136373 39867481 PMC11758362

[B13] Nelson EC (2011) Hardy heathers from the northern hemisphere: *Calluna*, *Daboecia*, *Erica*. Royal botanic gardens Kew, Richmond.

[B14] Nelson EC Small DJ (2000) International Register of Heather Names, Vol. 1: Hardy Cultivars and European Species (4 parts). The Heather Society, Creeting St Mary.

[B15] Nelson EC Small DJ (2004) International Register of Heather Names, Volume 2: African Species, Hybrids and Cultivars (4 parts). The Heather Society, Creeting St Mary. 10.5281/zenodo.10255788

[B16] Nelson EC Oliver EGH Pirie MD (2023) *Erica* L. (Ericaceae): Homonyms amongst published names for African species and proposed replacement names. PhytoKeys 236: 157–178. 10.3897/phytokeys.236.110498 38155765 PMC10753580

[B17] Nelson EC Pirie MD Bellstedt DU (2024) Redefining the megagenus *Erica* L. (Ericaceae): The contributions of E. G. H. Oliver and I. M. Oliver (née Nitzsche) to taxonomy and nomenclature. PhytoKeys 244: 39–55. 10.3897/phytokeys.244.121705 39006939 PMC11245645

[B18] Ojeda F (1998) Biogeography of seeder and resprouter *Erica* species in the Cape Floristic Region – Where are the resprouters? Biological Journal of the Linnean Society. Linnean Society of London 63: 331–347. 10.1111/j.1095-8312.1998.tb01521.x

[B19] Oliver EGH Linder HP Rourke JP (1983) Geographical distribution of present-day Cape taxa and their phytogeographical significance. Bothalia 14: 427–440. 10.4102/abc.v14i3/4.1189

[B20] Oliver EGH Forshaw N Oliver IM Volk F Schumann AWS Dorr LJ Hoekstra RD Musker SD Nürk NM Pirie MD Rebelo AG (2024) Genus *Erica*: An identification aid version 4.00. PhytoKeys 241: 143–154. 10.3897/phytokeys.241.117604 38699680 PMC11063621

[B21] Pasta S Zein HE Şentürk O Gücel S de Simone L de Montmollin B (2025) Broad-ranged, highly disjunct, locally rare and severely endangered: The challenging risk assessment and a global conservation strategy for *Ericasicula* Guss. sensu lato (Ericaceae). PhytoKeys 253: 295–329. 10.3897/phytokeys.253.121945 40135066 PMC11933908

[B22] Perrier de la Bathie H (1927) Les *Philippia* de Madagascar. Archives de botanique. Tome 1: 1–68. https://bibdigital.rjb.csic.es/records/item/12215-les-philippia-de-madagascar [September 5, 2024]

[B23] Pirie MD Blackhall-Miles R Bourke G Crowley D Ebrahim I Forest F Knaack M Koopman R Lansdowne A Nürk NM Osborne J Pearce TR Rohrauer D Smit M Wilman V (2022) Preventing species extinctions: A global conservation consortium for *Erica*. Plants, People, Planet 4: 335–344. 10.1002/ppp3.10266

[B24] Pirie MD Bellstedt DU Bouman RW Fagúndez J Gehrke B Kandziora M Maitre NCL Musker SD Newman E Nürk NM Oliver EGH Pipins S van der Niet T Forest F (2024) Spatial decoupling of taxon richness, phylogenetic diversity and threat status in the megagenus *Erica* (Ericaceae). PhytoKeys 244: 127–150. 10.3897/phytokeys.244.124565 39027483 PMC11255470

[B25] Raimondo D Van Staden L Foden W Victor JE Helme NA Turner RC Kamundi DA Manyama PA (2009) Strelitzia No. 25 Red list of South African plants 2009. South African National Biodiversity Institute, Pretoria, [ix +] 668 pp. http://hdl.handle.net/20.500.12143/260

[B26] Rebelo AG Siegfried WR Oliver EGH (1985) Pollination syndromes of Erica species in the south-western Cape. South African Journal of Botany 51: 270–280. 10.1016/S0254-6299(16)31657-X

[B27] Rodríguez-Buján I Díaz-Tapia P Fagúndez J (2024) Genetic and morphological evidence support the specific status of the endemic *Ericaandevalensis* (Ericales, Ericaceae). PhytoKeys 244: 57–76. 10.3897/phytokeys.244.120914 39006938 PMC11245639

[B28] Shaughnessy E (2024) E. Charles Nelson FLS, VMM (15 September 1951–20 May 2024). Archives of Natural History 51: 426–448. 10.3366/anh.2024.0939

[B29] Van der Niet T Cozien RJ (2024) Evidence for moth pollination in a rhinomyiophilous *Erica* species from the Cape Floristic Region of South Africa. PhytoKeys 246: 43–70. 10.3897/phytokeys.246.126310 39257487 PMC11384911

